# Risk Factors for Primary Middle East Respiratory Syndrome Coronavirus Illness in Humans, Saudi Arabia, 2014

**DOI:** 10.3201/eid2201.151340

**Published:** 2016-01

**Authors:** Basem M. Alraddadi, John T. Watson, Abdulatif Almarashi, Glen R. Abedi, Amal Turkistani, Musallam Sadran, Abeer Housa, Mohammad A. Almazroa, Naif Alraihan, Ayman Banjar, Eman Albalawi, Hanan Alhindi, Abdul Jamil Choudhry, Jonathan G. Meiman, Magdalena Paczkowski, Aaron Curns, Anthony Mounts, Daniel R. Feikin, Nina Marano, David L. Swerdlow, Susan I. Gerber, Rana Hajjeh, Tariq A. Madani

**Affiliations:** King Faisal Specialist Hospital and Research Centre, Jeddah, Saudi Arabia (B.M. Alraddadi);; Centers for Disease Control and Prevention, Atlanta, Georgia, USA (J.T. Watson, G.R. Abedi, J.G. Meiman, M. Paczkowski, A. Curns, A. Mounts, D.R. Feikin, N. Marano, D.L. Swerdlow, S.I. Gerber, R. Hajjeh);; Ministry of Health, Jeddah (A. Almarashi, A. Turkistani, A. Housa, A. Banjar, T.A. Madani);; Ministry of Health, Najran, Saudi Arabia (M. Sadran);; Ministry of Health, Riyadh, Saudi Arabia (M.A. Almazroa, N. Alraihan, A.J. Choudhry);; Ministry of Health, Alwajh, Saudi Arabia (E. Albalawi);; Ministry of Health, Hail, Saudi Arabia (H. Alhindi);; King Abdulaziz University, Jeddah (T.A. Madani)

**Keywords:** Middle East respiratory syndrome coronavirus, MERS-CoV, viruses, transmission, risk factors, primary infection, case–control study, dromedary camels, diabetes mellitus, heart disease, smoking, zoonosis, Saudi Arabia

## Abstract

Risk factors for primary Middle East respiratory syndrome coronavirus (MERS-CoV) illness in humans are incompletely understood. We identified all primary MERS-CoV cases reported in Saudi Arabia during March–November 2014 by excluding those with history of exposure to other cases of MERS-CoV or acute respiratory illness of unknown cause or exposure to healthcare settings within 14 days before illness onset. Using a case–control design, we assessed differences in underlying medical conditions and environmental exposures among primary case-patients and 2–4 controls matched by age, sex, and neighborhood. Using multivariable analysis, we found that direct exposure to dromedary camels during the 2 weeks before illness onset, as well as diabetes mellitus, heart disease, and smoking, were each independently associated with MERS-CoV illness. Further investigation is needed to better understand animal-to-human transmission of MERS-CoV.

## Abstract 2

Direct exposure to camels, diabetes mellitus, heart disease, and smoking were independently associated with this illness.

Middle East respiratory syndrome coronavirus (MERS-CoV) is a newly recognized respiratory pathogen first identified in a patient from Saudi Arabia in June 2012 (*1*). MERS-CoV causes acute respiratory disease that has a high case-fatality rate (*2*). All cases have been linked to countries in or near the Arabian Peninsula; >85% of cases have been reported from Saudi Arabia (*2*). Outbreaks of MERS-CoV have been associated primarily with transmission in healthcare settings (*3*–*5*). Transmission among household contacts of case-patients has been documented (*6*), but sustained human-to-human transmission has not (*7*). Low-level infections with MERS-CoV have been reported, but seroprevalence of MERS-CoV antibodies in the general population in Saudi Arabia is low (*8*). Strategies to prevent and control infection are recommended to limit secondary transmission in healthcare settings and among household contacts (*9*,*10*). MERS-CoV cases continue to be reported in Saudi Arabia in healthcare settings and in the community (*2*).

Animals have been suspected as a source of primary infection since early in the emergence of MERS-CoV, particularly given the similarities to severe acute respiratory syndrome coronavirus, a zoonosis known to cause human respiratory disease, often severe, with sustained human-to-human transmission and amplification in healthcare settings (*11*). Persons with early cases of MERS-CoV infection were observed to have had exposure to dromedary camels (henceforth dromedaries), and subsequent serologic studies from the Arabian Peninsula confirmed high seroprevalence of MERS-CoV neutralizing antibodies in dromedaries (*12*–*14*). Other studies have detected partial genome sequences of MERS-CoV from dromedary specimens (*15*–*17*), and more recently infectious MERS-CoV has been isolated from dromedaries (*16*,*18*–*21*). Additionally, a recent report provided virologic and serologic evidence of transmission of MERS-CoV from a sick dromedary to a human in Saudi Arabia (*19*).

Despite these reports, risk factors for primary illness with MERS-CoV (i.e., cases in persons without apparent exposure to other infected persons) are not well understood. No risk factors for primary transmission of MERS-CoV to humans have been confirmed by epidemiologic studies, including a link with exposure to dromedaries or any other animal species. We conducted a case–control study to assess exposures in primary cases and to identify risk factors associated with primary MERS-CoV illness in humans.

## Methods

### Study Design

In Saudi Arabia, all laboratory-confirmed MERS-CoV cases are reported to the Ministry of Health (MoH) and routinely investigated to assess preillness exposures. All cases reported during March 16–November 13, 2014, were screened for inclusion. For cases reported before May 13, 2014, a confirmed case was defined as illness in any person hospitalized with bilateral pneumonia and laboratory confirmation of MERS-CoV infection on the basis of a positive real-time reverse transcription PCR targeting 2 genes: the upstream of E gene and the open reading frame 1a gene (*22*). The case definition was revised on May 13, after which a confirmed case was defined as laboratory confirmation and any 1 of the following 4 clinical definitions: 1) fever and community-acquired pneumonia or acute respiratory distress syndrome based on clinical or radiologic evidence; 2) healthcare-associated pneumonia based on clinical and radiologic evidence in a hospitalized person; 3a) acute febrile (>38°C) illness, b) body aches, headache, diarrhea, or nausea/vomiting, with or without respiratory symptoms, and c) unexplained leucopenia (leukocytes <3.5 × 10^9^ cells/L) and thrombocytopenia (platelets <150 × 10^9^/L); 4) protected or unprotected exposure of a person (including a healthcare worker) to a confirmed or probable MERS-CoV infection and upper or lower respiratory illness within 2 weeks after exposure (*23*–*25*). For this study, case-patients were selected from among symptomatic patients whose illness met the case definition in place at the time of report and who met the study inclusion criteria described below.

### Case and Control Selection

Primary MERS-CoV cases were defined as cases in persons without known exposure to other MERS-CoV cases or recent (within 14 days) exposure to healthcare settings (*3*,*5*). MERS-CoV case-patients meeting this definition were presumed to have acquired infection through nonhuman contact. A trained MoH interviewer contacted the case-patient or proxy by phone or in person to conduct an initial screening. Case-patients were excluded if, within 14 days before onset of their MERS-CoV illness, they had been admitted to or visited any healthcare facility; had worked in a healthcare facility in any capacity; had a recognized epidemiologic link with another person either with confirmed MERS-CoV infection or with an acute respiratory illness (as perceived by the participant) of unknown cause; were <18 years of age; or did not provide consent for interview either personally or by proxy (i.e., a family member or close friend familiar with the preillness activities and usual habits of the case-patient) for case-patients who had died or were too ill to give consent personally.

For each case-patient, we randomly selected up to 4 neighborhood controls matched by age and sex. For case-patients 18 to <25 years old, controls were matched within 5 years of age, and for those >25 years old, controls were matched within 10 years of age. First, starting at the case-patient’s household, a random direction was selected by flipping a coin. Second, the distance in number of houses from the case-patient’s residence was randomly determined from 1 to 10 by choosing from a random number list. For multifamily structures, the starting floor and apartment were randomly chosen. Once a household was identified, 1 control was selected on the basis of the matching criteria; the exclusion criteria used for case-patients were also applied for all controls. If >1 person in the household met matching criteria, 1 was randomly chosen. If no matching control was found in the selected household, the next house in the same direction was visited, and so on, until an eligible control was enrolled.

### Interview Process

A case–control protocol developed by the World Health Organization was adapted to create a standardized questionnaire for assessing risk factors associated with MERS-CoV illness. This questionnaire was used by MoH staff to conduct in-person interviews with case-patients (or their proxies) and controls (*26*). The 14-day period before illness onset was defined as the exposure period both for case-patients and their corresponding controls.

### Data Collection

The questionnaire addressed demographic information; medical history; travel history; and information about human, food, and animal exposures. Human exposure questions addressed preillness exposures to healthcare settings or persons with acute respiratory illness. Food exposure questions assessed consumption of fruit, vegetables, unpasteurized milk, meats, urine, or chewing of siwak (a twig from the *Salvadora persica* tree, traditionally used for teeth cleaning). Animal exposure questions addressed multiple species (dromedaries, goats, sheep, horses, cattle) and whether any direct or indirect exposure to animals occurred. Direct animal exposure in the 14-days before illness onset was defined as physical contact with animals or animal products (carcasses, body fluids, secretions, urine, excrement, or raw meat) in any setting (farm, livestock market, slaughterhouse, racetrack, stable, or other animal-related venue) or engaging in certain animal-related activities (feeding animals, cleaning housing, slaughtering, assisting with birth, milking, kissing or hugging, or other related tasks). Indirect animal exposure in the 14 days before illness onset was defined as having visited settings where animals were kept but without having direct contact; or exposure to household members who themselves had direct animal exposure. When assessing animal exposure during the previous 6 months, participants self-defined direct physical contact. Interviews with case-patients and controls were conducted in Arabic or English. 

Because this investigation was part of a public health response, MoH and the US Centers for Disease Control and Prevention (CDC; Atlanta, GA, USA) determined it to be nonresearch and therefore not subject to institutional review board review. We obtained written informed consent from all participants or their proxies.

### Statistical Analysis

We used Epi Info 7 (CDC, Atlanta GA, USA) for data entry and SAS version 9.3 (SAS Institute Inc., Cary, NC, USA) for data analysis. Characteristics that were not part of the matching process for case-patients and controls were compared using χ^2^ tests, Fisher exact tests, or *t* tests. We used exact conditional logistic regression to estimate odds ratios (ORs), 95% mid-p CIs, and exact p values for potential risk factors for MERS-CoV illness. Factors found to be significant (p<0.05) in the univariate analysis were further evaluated in multivariable analyses. We created a final multivariable model through stepwise elimination of nonsignificant variables until all remaining variables in the multivariate model were significant at p<0.05. Interactions between risk factors were also evaluated in the multivariable analyses.

## Results

During March 16–November 13, 2014, a total of 535 patients with laboratory-confirmed MERS-CoV infection were reported to the MoH. After screening based on the exclusion criteria, 34 patients were identified as possible primary case-patients. Two persons refused to participate, and 2 did not meet the age criteria for inclusion. The remaining 30 case-patients, representing 8 of 13 regions in Saudi Arabia, were enrolled in the study (Figure). Symptom onset dates for enrolled case-patients ranged from February 25 through November 2, 2014.

**Figure:**
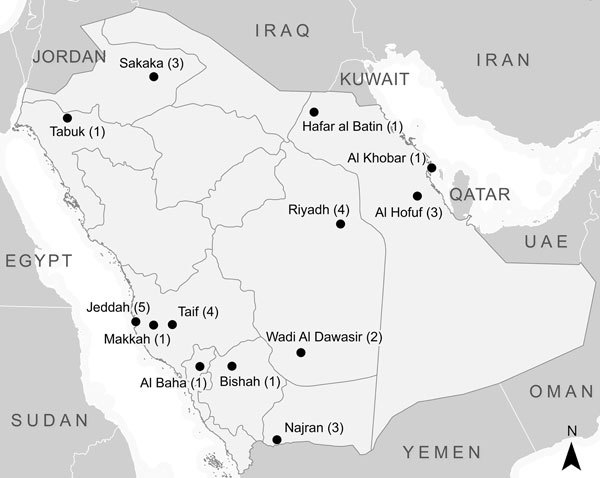
City or governorate of residence of persons with primary Middle East respiratory syndrome coronavirus included in the study, Saudi Arabia, March 16–November 13, 2014.

We identified and included 116 controls: 4 age-, sex- and neighborhood-matched controls for each of 28 case-patients and 2 controls for each of the 2 remaining case-patients. Of the 116 controls, ages for 6 exceeded the age-matching criteria by 1–5 years. One case-patient did not have a formal residence; after his interview, 4 controls were selected from the vicinity of the area where he spent his nights. 

Case-patients and controls were interviewed during June 8–November 29, 2014. Interviews with case-patients and controls were conducted on the same day, a median of 27 days (range 0–192 days, interquartile range 12–70 days) after case-patients’ illness onsets.

Median age of case-patients was 49 years; 97% were male (Table 1). Rate of ownership of a barn or farm with animals was similar between case-patients and controls, but income was higher for case-patients than for controls (53% vs. 34%, respectively, had a monthly income of >6,000 SAR [US $1,600]; 2013 gross national income per capita is $2,188/month in Saudi Arabia [*27*]). Other demographic features between the groups were similar. At the time of interview, 7 (23%) case-patients were deceased, and 10 (33%) were too ill to be interviewed. For these 17 (57%) case-patients and for 1 (1%) control, a family member served as proxy. Case-patients interviewed by proxy were more likely than those interviewed directly to have underlying medical conditions but were similar in age, other demographic characteristics, and animal-related exposures.

**Table 1:** Demographic characteristics of Middle East respiratory syndrome coronavirus case-patients compared with age- and sex-matched neighborhood controls. Saudi Arabia, March 16–November 13, 2014

Variable*	Total, no. (%), n = 146	Case-patients, no. (%), n = 30	Controls, no. (%), n = 116	p value
Sex				1.000†
F	5 (3)	1 (3)	4 (3)	
M	141 (97)	29 (97)	112 (97)	
Interview respondent‡				<0.001†
Self	128 (88)	13 (43)	115 (99)	
Proxy (relative)	18 (12)	17 (57)	1 (1)	
Nationality				0.620§
Saudi	98 (67)	19 (63)	79 (68)	
Non-Saudi	48 (33)	11 (37)	37 (32)	
Education				0.850§
Primary school or less	65 (45)	14 (47)	51 (44)	
More than primary school	81 (55)	16 (53)	65 (56)	
Household income (monthly)‡				0.047§
<6,000 SAR	91 (62)	14 (47)	77 (66)	
>6,000 SAR	55 (38)	16 (53)	39 (34)	
Marital status				0.475§
Never married	8 (5)	3 (10)	5 (4)	
Married	133 (91)	26 (87)	107 (92)	
Widowed	5 (3)	1 (3)	4 (3)	

*Median ages (ranges) in years are as follows: case-patients, 49 (20–72); controls, 50 (19–74); all, 50 (19–74). p<0.846, pooled t test. †Fisher exact test. ‡Statistically significant (p<0.05). §χ^2^ test.

Several exposures were associated with MERS-CoV by univariate analysis (Table 2). During the 14 days before illness onset, case-patients were more likely than controls to have had direct dromedary exposure (33% vs. 15%, OR 3.73, 95% CI 1.24–11.80); to keep dromedaries in or around the home (30% vs. 15%, OR 3.34, 95% CI 1.04–10.98); or to have visited a farm where dromedaries were present (90% vs. 53%, OR 11.57, 95% CI 2.67–∞); Among those who visited a farm where livestock were kept during the exposure period, case-patients were more likely than controls to have milked dromedaries (50% vs. 23%, OR 10.36, 95% CI 2.47–∞). Case-patients also were more likely than controls to live in the same household as someone who had visited a farm with dromedaries during the previous 14 days (30% vs. 12%, OR 3.95, 95% CI 1.23–13.72) and to have had direct contact with a dromedary while there (40% vs. 15%, OR 5.03, 95% CI 1.66–16.88). Case-patients also were more likely than controls to have had direct physical contact with dromedaries in the previous 6 months (37% vs. 13%, OR 7.67, 95% CI 2.10–36.08). Case-patients were no more likely than controls to report exposure to bats, goats, horses, sheep, or the products of these animals; however, direct cattle exposure was significantly associated with illness (13% vs. 3%, OR 6.00, 95% CI 1.02–48.44). No differences were noted in consumption of fruits; vegetables; or animal products, including uncooked meat, unpasteurized animal milk, or dromedary urine (Technical Appendix Table 1). We observed no significant differences in dromedary exposures between case-patients interviewed directly and those interviewed by proxy.

**Table 2:** Animal-related exposures, underlying health conditions, current tobacco use, and siwak use for Middle East respiratory syndrome coronavirus case-patients compared with matched controls. Saudi Arabia, March 16–November 13, 2014

Variable	No. (%) with exposure*		Odds ratio (95% CI)	p value
	Case-patients, n = 30	Controls, n = 116		
Animal-related exposures				
Household members frequently visit farms with dromedaries†‡	12/30 (40)	14/115 (12)	7.06 (2.23–26.46)	<0.001
Household members visited a farm with dromedaries during exposure period‡	9/30 (30)	14/115 (12)	3.95 (1.23–13.72)	0.018
Household members had direct contact with dromedaries during exposure period‡§	12/30 (40)	17/114 (15)	5.03 (1.66–16.88)	0.004
Spouse	4/30 (13)	4/116 (3)	4.26 (0.86–23.41)	0.065
Other relatives‡	7/30 (23)	7/116 (6)	4.59 (1.36–16.27)	0.012
Domestic help‡	5/30 (17)	3/116 (3)	15.04 (1.96−369.59)	0.006
Dromedaries kept in/around home during exposure period‡§	9/30 (30)	17/115 (15)	3.34 (1.04–10.98)	0.047
Goats kept in/around home during exposure period‡§	1/30 (3)	22/115 (19)	0.08 (0.003–0.58)	0.011
Horses kept in/around home during exposure period§	1/29 (3)	0/115 (0)	4.00 (0.44–∞)	0.200
Bats in/around house during exposure period§	3/28 (11)	11/112 (10)	1.60 (0.24–9.23)	0.646
Sheep kept in/around home during exposure period§	10/30 (33)	22/115 (19)	3.34 (0.97–12.19)	0.057
Sheep present at a slaughterhouse visited during exposure period‡§	1/30 (3)	18/116 (16)	0.15 (<0.001–0.56)	0.040
Visited farm where livestock were kept during exposure period§	10/29 (34)	32/116 (28)	1.67 (0.52–5.42)	0.393
Dromedary present on farm‡	9/10 (90)	17/32 (53)	11.57 (2.67–∞)	0.013
Milked dromedaries while on farm‡	5/10 (50)	7/31 (23)	10.36 (2.47–∞)	0.013
Visited other livestock venue (i.e., not farm, market, slaughterhouse, racetrack, or stable) during exposure period‡§	7/29 (24)	12/111 (11)	3.33 (1.001–11.05)	0.040
Direct physical contact with dromedary during last 6 mo‡	11/30 (37)	15/116 (13)	7.67 (2.10–36.08)	0.001
Any direct contact with a dromedary during exposure period‡§¶	10/30 (33)	17/116 (15)	3.73 (1.24–11.80)	0.020
Any direct contact with a goat during exposure period§	4/30 (13)	22/116 (19)	0.64 (0.17–2.02)	0.584
Any direct contact with a§ sheep during exposure period§¶	10/30 (33)	38/116 (33)	1.03 (0.37–2.77)	1.000
Any direct contact with a horse during exposure period§¶	1/30 (3)	0/116 (0)	4.00 (0.44–∞)	0.200
Any direct contact with cattle during exposure period§¶	4/30 (13)	4/116 (3)	6.00 (1.02–48.44)	0.043
Underlying health conditions and behaviors				
Diabetes‡	16/29 (55)	32/116 (28)	3.72 (1.45–10.25)	0.005
Emphysema, chronic bronchitis, or other chronic lung disease‡	4/30 (13)	1/113 (1)	17.68 (4.22-∞)	0.003
Heart disease‡	11/30 (37)	14/114 (12)	5.11 (1.81–15.46)	0.002
Current smoker‡	11/30 (37)	22/116 (19)	3.14 (1.10–9.24)	0.030
Any underlying condition‡#	21/30 (70)	49/116 (42)	5.11 (1.70–18.67)	0.004
Any underlying condition, including current smoking‡	27/30 (90)	64/116 (55)	7.55 (2.32–33.45)	<0.001
Using *siwak* during exposure period‡§	7/28 (25)	56/114 (49)	0.24 (0.06–0.77)	0.023

*Denominators vary on the basis of completeness of responses or reflect subsets. †Dromedaries, dromedary camels. ‡Statistically significant (p<0.05). §The exposure period of cases is defined as the 14 days before the date of the first symptom onset. For controls, the exposure period is the same as for the case to which they are matched. ¶Direct animal contact includes any of the following specific exposures: physical contact with animals or animal products (i.e., carcasses, body fluids, secretions, urine, excrement, or raw meat) in any setting (i.e., farm, livestock market, slaughterhouse, racetrack or stable, or other animal-related venues) or engaging in certain animal-related activities (i.e., feeding animals, cleaning their housing, slaughtering them, assisting with their birth, milking them, kissing or hugging them, or other related tasks). #Diabetes, asthma, emphysema, chronic bronchitis, other chronic lung disease, kidney failure, chronic liver disease, heart disease, history of cancer treatment, blood disorder.

Case-patients were more likely than controls to have >1 underlying medical condition (70% vs. 42%, OR 5.11, 95% CI 1.70–18.67). Diabetes mellitus (55% vs. 28%, OR 3.72, 95% CI 1.45–10.25); heart disease (37% vs. 12%, OR 5.11, 95% CI 1.81–15.46); and chronic lung disease (13% vs. 1%, OR 17.68, 95% CI 4.22–∞) were each reported significantly more frequently among case-patients than among controls. No significant differences were identified in other reported health conditions (asthma, kidney failure, chronic liver disease, cancer, blood disorders, or conditions requiring corticosteroid use). Case-patients also were more likely than controls to currently smoke tobacco (37% vs. 19%, OR 3.14, 95% CI 1.10–9.24). Using siwak during the exposure period was associated with a lower risk for MERS-CoV illness (25% vs. 49%, OR 0.24, 95% CI, 0.06–0.77).

Multivariable analysis yielded a final model in which direct dromedary exposure in the 2 weeks before illness onset was associated with MERS-CoV illness (adjusted OR 7.45, 95% CI 1.57–35.28), along with having diabetes (adjusted OR 6.99, 95% CI 1.89–25.86) or heart disease (adjusted OR 6.87, 95% CI 1.81–25.99) or currently smoking tobacco (adjusted OR 6.84, 95% CI 1.68–27.94) (Technical Appendix Table 2). When substituting direct physical contact with dromedaries in the previous 6 months for direct dromedary exposure in the past 2 weeks, we found this exposure to be significantly associated with MERS-CoV illness (adjusted OR 14.59, 95% CI 2.38–89.55) along with previously identified risk factors: having diabetes (adjusted OR 6.95, 95% CI 1.85–26.12) or heart disease (adjusted OR 6.09, 95% CI 1.61–22.94) or currently smoking tobacco (adjusted OR 7.36, 95% CI 1.75–30.94). We identified no significant interactions for direct dromedary exposure, having diabetes, having heart disease, or currently smoking tobacco and other exposures, underlying conditions, or behaviors.

## Discussion

By carefully identifying persons with primary MERS-CoV infections and systematically comparing their characteristics to age- and sex-matched neighborhood controls, our study supports a link between exposure to dromedaries and human MERS-CoV illness, as well as host risk factors (i.e., diabetes, heart disease, and smoking). Exposure to bats, goats, horses, sheep, or the products of these animals were not associated with MERS CoV illness in our study. The role of an animal reservoir in the transmission of MERS-CoV to humans has been actively considered since the first reported cases in 2012. Our investigation was designed to broadly assess the possible routes and modes of transmission of MERS-CoV and to determine the risk associated with exposure to different animal species and general environmental factors.

In our study, direct contact with dromedaries in the 2 weeks before illness onset was associated with MERS-CoV illness. The proportions reporting direct contact with dromedaries was limited among both case-patients and controls (33% vs. 15%). Among specific direct exposures that we investigated, only milking dromedaries was significantly associated with illness. However, we noted a significant association when considering together all reported activities that involve direct dromedary exposure. When we controlled for underlying conditions, direct exposure to dromedaries (whether in the previous 2 weeks or in the previous 6 months) remained an independent risk factor for MERS-CoV illness. Additionally, living in the same household with persons who reported working on or visiting a farm where dromedaries were kept was a risk factor for illness; although the numbers were small, the highest risks were associated with other relatives and domestic helpers. Indirect contact with dromedaries might explain primary MERS-CoV illness in case-patients without direct dromedary contact and should be further explored. Other potential explanations of MERS-CoV illness in primary case-patients who did not have direct contact with dromedaries include unrecognized community exposure to patients with mild or subclinical MERS-CoV infection or exposure to other sources of primary MERS-CoV infection not ascertained in our study. A recent nationwide serosurvey from Saudi Arabia estimated that >44,000 persons might be seropositive for MERS-CoV and might be the source of infection to patients with confirmed primary MERS-CoV illness but with no dromedary exposure (*8*). Although we found that direct and indirect dromedary exposure were significantly associated with MERS-CoV illness, our study had limited power to detect specific behaviors or practices associated with illness. Future studies should be designed to further explore this association.

Case-patients in our study were significantly more likely than controls to report diabetes; this finding provides epidemiologic evidence of diabetes as a risk factor for MERS-CoV illness. Smoking and heart disease were also significantly associated with MERS-CoV illness. Of note was the overwhelming male preponderance in our study; only 1 of the 30 case-patients with primary infection was female. The fact that men in Saudi Arabia are much more likely than women to have contact with dromedaries might explain this observation. Previous studies have reported some male preponderance, but those findings were not as striking as our results, probably because MERS-CoV infections in most patients in other studies were healthcare associated and transmitted from human to human (*2*,*4*)

Our study is subject to several limitations. First, the delay between illness and interview might have affected recall among study participants. Second, our study was a nationwide investigation that covered a large area, and interviews were conducted by different teams at different times. However, all interviewers received training designed to limit interview variability. Third, MERS-CoV is a highly lethal disease, and 17 of the 30 case-patients in our study were interviewed by proxy (i.e., information was collected from a family member), which might have affected the reliability of the exposure information collected. Fourth, choosing neighborhood controls could have resulted in an underestimation of certain risk factors because of possible similarities between case-patients and controls. However, the study still identified an association with dromedary exposure. Fifth, the occurrence of primary MERS-CoV cases is a relatively rare event, limiting the number of cases available for inclusion in our study and the power to detect differences from controls. We applied stringent criteria for enrollment and attempted to exclude persons who might have acquired infection through human-to-human transmission, but the possibility of misclassification remains. However, inadvertent inclusion of secondary cases is likely to mean that the true risk associated with dromedary exposure was higher than we estimated. Sixth, we did not investigate dromedary husbandry practices or ascertain whether dromedaries were infected with MERS-CoV. Additionally, the surveillance system in place in Saudi Arabia might be more likely to detect persons severely affected by MERS-CoV, who also might be more likely to have underlying conditions. This fact might have overstated the role of underlying conditions as a risk factor for disease. Finally, as in any study where a large number of parameters are tested, the expected type 1 error rate is 5%; therefore, one could anticipate that 1 in 20 significant results would incorrectly reject the null hypothesis.

In conclusion, our findings represent an important initial step in understanding the risk factors for MERS-CoV infection, including zoonotic transmission. Control of MERS-CoV ultimately depends on the interruption of transmission to prevent primary MERS-CoV cases. Future longitudinal studies to assess specific human–dromedary interactions are needed to inform preventive measures.

Technical Appendix All findings from conditional logistic regression of Middle East respiratory syndrome coronavirus cases and matched controls and selected demographic, exposure, and underlying condition information for case-patients who had any direct contact with dromedary camels.
