# Supplementary material for: Risk Factors for Primary Middle East Respiratory Syndrome Coronavirus Illness in Humans, Saudi Arabia, 2014
Source: Emerg Infect Dis. 2016 Jan;22(1):49–55. doi: 10.3201/eid2201.151340 (PMC4696714; doi:10.3201/eid2201.151340)
Supplement: Technical Appendix — All findings from conditional logistic regression of Middle East respiratory syndrome coronavirus cases and matched controls and selected demographic, exposure, and underlying condition information for case-patients who had any direct contact with dromedary camels. [file 15-1340-Techapp-s1.pdf]

Article DOI: http://dx.doi.org/10.3201/eid2201.151340

Risk Factors for Primary MERS-CoV Illness in Humans, Saudi Arabia, 2014

Technical Appendix

Technical Appendix Table 1. All findings from conditional logistic regression of Middle East respiratory syndrome coronavirus case-patients and matched controls, Saudi Arabia, March 16–November 13, 2014

Exposure	Case-patients, no. (%), n = 30	Controls, no. (%), n = 116	Odds ratio (95% CI)	p value
Travel history				
Traveled outside the country during the exposure period*	0/30 (0)	1/116 (1)	4.0 (<0.001–36.0)	1.000
Traveled within the country during the exposure period*	8/30 (27)	20/115 (17)	1.9 (0.6–5.3)	0.271
Attended recent mass gatherings within the country during the exposure period*	3/30 (10)	11/111 (10)	1.0 (0.2–4.0)	1.000
Animal exposures				
Owned farm/barn with animals	11/30 (37)	39/116 (34)	1.4 (0.4–4.3)	0.773
Household members frequently visited farms with camels	12/30 (40)	14/115 (12)	7.1 (2.2–26.5)	0.001
Others in household visited a farm during exposure period*	9/30 (30)	14/115 (12)	3.9 (1.2–13.7)	0.018
Others in household had direct contact with camels during exposure period*	12/30 (40)	17/114 (15)	5.0 (1.7–16.9)	0.004
Spouse	4/30 (13)	4/116 (3)	4.3 (0.9–23.4)	0.065
Relative	7/30 (23)	7/116 (6)	4.6 (1.4–16.3)	0.012
Domestic help	5/30 (17)	3/116 (3)	15.0 (2.0–369.6)	0.006
Other resident	2/30 (7)	4/116 (3)	2.2 (0.2–68.6)	0.600
Livestock kept in/around home during exposure period*	12/30 (40)	38/115 (33)	1.6 (0.5–4.9)	0.390
Bats in/around home during exposure period*	3/28 (11)	11/112 (10)	1.6 (0.2–9.2)	0.646
Other animals in/around home during exposure period*	12/28 (43)	32/106 (30)	2.0 (0.8–5.5)	0.203
Visited a farm where livestock were kept during exposure period*	10/29 (34)	32/116 (28)	1.7 (0.5–5.4)	0.393
Camels kept at home	9/30 (30)	17/115 (15)	3.3 (1.0–11.0)	0.047
Goats kept at home	1/30 (3)	22/115 (19)	0.1 (0.0–0.6)	0.011
Sheep kept at home	10/30 (33)	22/115 (19)	3.3 (1.0–12.2)	0.057
Horses kept at home	1/29 (3)	0/115 (0)	4.0 (0.4–∞)	0.200
Visited a farm where livestock were kept during exposure period*	10/29 (34)	32/116 (28)	1.7 (0.5–5.4)	0.393
Camel present on farm	9/10 (90)	17/32 (53)	11.6 (2.7–∞)	0.013
Goat present on farm	3/10 (30)	20/32 (63)	0.5 (0.1–4.2)	0.648
Sheep present on farm	6/10 (60)	24/32 (75)	0.4 (0.0–3.1)	0.627
Physical contact with livestock	9/10 (90)	20/32 (63)	4.2 (0.6–103.9)	0.220
Any animals sick	2/8 (25)	5/31 (16)	1.7 (0.0–77.5)	1.000
Ate or drank anything while on farm	6/10 (60)	14/30 (47)	0.6 (0.0–5.4)	1.000
Touched items that may have had contact with animals	6/10 (60)	17/32 (53)	1.7 (0.3–15.2)	0.663
Contact with animal carcasses, body fluids, secretions, urine, or excrement	3/10 (30)	9/32 (28)	0.4 (<0.001–2.0)	0.292
Contact with animal bedding or feed	4/10 (40)	14/32 (44)	0.4 (0.0–4.4)	0.625
Fed animals	6/10 (60)	16/31 (52)	1.7 (0.3–15.2)	0.663
Cleaned animal housing	3/9 (33)	3/31 (10)	1.4 (0.0–58.3)	1.000
Cleaned farm equipment	2/10 (20)	2/31 (6)	1.0 (0.1–∞)	1.000
Slaughtered animals	2/9 (22)	12/31 (39)	0.7 (<0.001–4.0)	0.500
Assisted with animal birth	1/9 (11)	6/31 (19)	0.5 (<0.001–2.4)	0.525
Milked camels	5/10 (50)	7/31 (23)	10.4 (2.5–∞)	0.013
Kissed/hugged camels	1/9 (11)	7/31 (23)	0.7 (0.0–7.0)	1.000

Exposure	Case-patients, no. (%), n = 30	Controls, no. (%), n = 116	Odds ratio (95% CI)	p value
Other tasks	0/8 (0)	2/30 (7)	1.0 (<0.001–9.0)	1.000
Aware of bats in/around farm	2/8 (25)	9/31 (29)	0.3 (0.0–5.1)	0.533
Visited market selling livestock animals during exposure period*	8/28 (29)	24/115 (21)	1.8 (0.5–6.2)	0.345
Camel present at market	4/10 (40)	6/25 (24)	2.3 (0.3–20.4)	0.634
Goat present at market	3/10 (30)	10/25 (40)	0.8 (0.0–12.6)	1.000
Sheep present at market	4/10 (40)	19/25 (76)	0.2 (0.0–1.4)	0.164
Direct physical contact with any animals	7/8 (88)	18/24 (75)	1.0 (0.0–63.9)	1.000
Direct contact with camels	3/10 (30)	5/25 (20)	1.6 (0.1–17.0)	1.000
Direct contact with goats	2/10 (20)	8/25 (32)	0.5 (<0.001–4.5)	0.333
Direct contact with sheep	5/10 (50)	16/25 (64)	0.3 (0.0–2.1)	0.359
Consumed food at market	2/7 (29)	5/23 (22)	1.7 (0.1–58.5)	1.000
Visited slaughterhouse during exposure period*	2/28 (7)	22/114 (19)	0.2 (0.0–1.0)	0.100
Camel present at slaughterhouse	1/4 (25)	7/24 (29)	2.0 (0.1–78.0)	1.000
Goat present at slaughterhouse	0/4 (0)	12/24 (50)	0.4 (<0.001–1.8)	0.2778
Sheep present at slaughterhouse	1/30 (3)	18/116 (16)	0.2 (<0.001–0.6)	0.040
Direct physical contact with any animals	1/2 (50)	8/20 (40)	1.0 (0.0–48.3)	1.000
Direct contact with camels	0/4 (0)	3/24 (13)	–	–
Direct contact with goats	0/4 (0)	3/24 (13)	2.0 (<0.001–18.0)	1.000
Direct contact with sheep	1/4 (25)	7/24 (29)	0.5 (0.0–6.9)	1.000
Visited racetrack/stable where camels were present during exposure period*	4/30 (13)	9/115 (8)	2.6 (0.5–12.9)	0.355
Direct contact with camels	3/3 (100)	4/8 (50)	0.3 (0.0–∞)	1.000
Visited other livestock venue (i.e., not farm, market, slaughterhouse, racetrack, or stable) during exposure period*	7/29 (24)	12/111 (11)	3.3 (1.0–11.1)	0.040
Direct contact with any animals	4/6 (67)	8/12 (67)	0.3 (0.0–∞)	1.000
Direct contact with camels	4/8 (50)	7/17 (41)	0.3 (0.0–∞)	1.000
Direct contact with goats	1/8 (13)	6/17 (35)	0.3 (<0.001–1.8)	0.200
Direct contact with sheep	3/8 (38)	6/17 (35)	1.2 (0.0–48.7)	1.000
Slaughtered animal during exposure period*	3/28 (11)	16/114 (14)	0.6 (0.1–2.3)	0.510
Slaughtered camel	2/30 (7)	1/116 (1)	6.5 (0.5–196.4)	0.147
Slaughtered goat	1/30 (3)	3/116 (3)	1.3 (0.1–12.5)	1.000
Slaughtered sheep	3/30 (10)	11/116 (9)	1.1 (0.2–5.1)	1.000
Direct contact with camels in last 6 months	11/30 (37)	15/116 (13)	7.7 (2.1–36.1)	0.001
Direct contact with goats in last 6 months	2/30 (7)	24/116 (21)	0.2 (0.0–1.0)	0.081
Direct contact with sheep in last 6 months	10/30 (33)	34/116 (29)	1.3 (0.5–3.7)	0.796
Visited any venue where camels were present during exposure period*	12/30 (40)	29/116 (25)	2.3 (0.8–6.3)	0.111
Visited any venue where goats were present during exposure period*	6/30 (20)	35/116 (30)	0.5 (0.2–1.5)	0.306
Visited any venue where sheep were present during exposure period*	13/30 (43)	46/116 (40)	1.1 (0.4–3.0)	1.000
Visited any venue where horses were present during exposure period*	1/30 (3)	0/116 (0)	4.0 (0.4–∞)	0.200
Visited any venue where cattle were present during exposure period*	1/30 (3)	5/116 (4)	0.8 (0.0–6.5)	1.000
Any direct contact with a camel during exposure period*†	10/30 (33)	17/116 (15)	3.7 (1.2–11.8)	0.020
Any direct contact with a goat during exposure period*†	4/30 (13)	22/116 (19)	0.6 (0.2–2.0)	0.584
Any direct contact with a sheep during exposure period*†	10/30 (33)	38/116 (33)	1.0 (0.4–2.8)	1.000
Any direct contact with a horse during exposure period*†	1/30 (3)	0/116 (0)	4.0 (0.4–∞)	0.200
Any direct contact with a cattle during exposure period*†	4/30 (13)	4/116 (3)	6.0 (1.0–48.4)	0.043
Any indirect contact with a camel during exposure period*‡	4/30 (13)	16/116 (14)	0.8 (0.2–3.0)	1.000
Any indirect contact with a goat during exposure period*‡	2/30 (7)	15/116 (13)	0.5 (0.1–2.0)	0.364
Any indirect contact with a sheep during exposure period*‡	3/30 (10)	15/116 (0)	0.5 (0.1–2.4)	0.503
Any indirect contact with a horse during exposure period*‡	0/30 (0)	#REF!	0.5 (0.1–2.4)	0.503
Any indirect contact with a cow during exposure period*‡	0/30 (0)	4/116 (3)	0.8 (<0.001–3.1)	0.590
Direct or indirect contact with a camel during exposure period*†‡	14/30 (47)	33/116 (28)	2.7 (1.0–7.5)	0.070
Direct or indirect contact with a goat during exposure period*†‡	6/30 (20)	37/116 (32)	0.5 (0.1–1.3)	0.208
Direct or indirect contact with a sheep during exposure period*†‡	13/30 (43)	53/116 (46)	0.8 (0.3–2.1)	0.802
Direct or indirect contact with a horse during exposure period*†‡	1/30 (3)	0/116 (0)	4.0 (0.4–∞)	0.200
Direct or indirect contact with a cow during exposure period*†‡	4/30 (13)	8/116 (7)	2.4 (0.5–9.9)	0.237

Exposure	Case-patients, no. (%), n = 30	Controls, no. (%), n = 116	Odds ratio (95% CI)	p value
Food exposures				
Ate raw dried fruits during exposure period*	3/29 (10)	11/116 (9)	1.1 (0.2–3.8)	1.000
Ate raw dates during exposure period*	17/28 (61)	77/116 (66)	0.8 (0.3–2.2)	0.796
Ate raw vegetables during exposure period*	22/29 (76)	97/116 (84)	0.5 (0.2–1.6)	0.354
Ate uncooked/partially cooked meat during exposure period*	2/29 (7)	5/116 (4)	1.6 (0.2–8.1)	0.633
Cooked/handled raw meat during exposure period*	8/28 (29)	28/112 (25)	1.1 (0.4–3.6)	1.000
Drank camel urine during exposure period*	2/28 (7)	3/114 (3)	2.3 (0.3–15.8)	0.590
Used siwak during exposure period*	7/28 (25)	56/114 (49)	0.2 (0.1–0.8)	0.023
Exposed to sandstorms during study period*	15/24 (63)	60/107 (56)	3.3 (0.4–88.7)	0.363
Handled raw camel meat during exposure period*	1/30 (3)	4/116 (3)	0.8 (0.0–8.3)	1.000
Handled raw goat meat during exposure period*	0/30 (0)	2/116 (2)	1.7 (<0.001–8.6)	1.000
Handled raw sheep meat during exposure period*	3/30 (10)	21/116 (18)	0.4 (0.1–1.6)	0.248
Handled raw cattle meat during exposure period*	3/30 (10)	4/116 (3)	4.2 (0.6–36.7)	0.131
Ate raw camel meat during exposure period*	0/30 (0)	0/116 (0)	4.2 (0.6–36.7)	0.131
Ate raw goat meat during exposure period*	0/30 (0)	0/116 (0)	4.2 (0.6–36.7)	0.131
Ate raw sheep meat during exposure period*	1/30 (3)	1/116 (1)	4.0 (0.1–156.0)	0.360
Ate raw cattle meat during exposure period*	1/30 (3)	0/116 (0)	4.0 (0.4–∞)	0.200
Drank unpasteurized camel milk during exposure period*	6/30 (20)	17/116 (15)	1.7 (0.4–8.1)	0.708
Drank unpasteurized goat milk during exposure period*	0/30 (0)	5/116 (4)	0.5 (<0.001–2.3)	0.565
Drank unpasteurized sheep milk during exposure period*	0/30 (0)	2/116 (2)	1.7 (<0.001–8.6)	1.000
Drank unpasteurized cattle milk during exposure period*	0/30 (0)	1/116 (1)	4.0 (<0.001–36.0)	1.000
Consumed raw/ unpasteurized camel meat/milk during exposure period*	6/30 (20)	20/116 (17)	1.2 (0.3–4.9)	1.000
Consumed raw/ unpasteurized goat meat/milk during exposure period*	0/30 (0)	5/116 (4)	0.5 (<0.001–2.3)	0.565
Consumed raw/unpasteurized sheep meat/milk during exposure period*	1/30 (3)	3/116 (3)	1.4 (0.0–19.8)	1.000
Consumed raw/unpasteurized cattle meat/milk during exposure period*	1/30 (3)	1/116 (1)	4.0 (0.1–156.0)	0.360
Background medical history				
Diabetes	16/29 (55)	32/116 (28)	3.7 (1.5–10.3)	0.005
Asthma	4/29 (14)	4/116 (3)	4.3 (0.8–24.1)	0.072
Emphysema, chronic bronchitis, or other chronic lung disease	4/30 (13)	1/113 (1)	17.7 (4.2–∞)	0.003
Kidney failure	1/30 (3)	1/114 (1)	4.0 (0.1–156.0)	0.360
Chronic liver disease	1/30 (3)	2/113 (2)	–	–
Blood disorder	2/29 (7)	1/114 (1)	5.1 (0.4–157.0)	0.200
Heart disease	11/30 (37)	14/114 (12)	5.1 (1.8–15.5)	0.002
Cancer treatment in last year	1/30 (3)	2/114 (2)	2.0 (0.1–26.3)	1.000
Was taking corticosteroids	2/30 (7)	4/116 (3)	1.8 (0.2–10.1)	0.618
Used traditional medications during exposure period*	1/30 (3)	3/114 (3)	1.3 (0.1–12.5)	1.000
Current smoker	11/30 (37)	22/116 (19)	3.1 (1.1–9.2)	0.030
Past smoker	13/29 (45)	40/112 (36)	1.7 (0.6–4.5)	0.325
Any underlying condition, excluding current smoking	21/30 (70)	49/116 (42)	5.1 (1.7–18.7)	0.004
Any underlying condition, including current smoking	27/30 (90)	64/116 (55)	7.6 (2.3–33.4)	<0.001

*The exposure period of cases is defined as the 14 days before the date of the first symptom onset. For controls, the exposure period is the same as that of the case-patient to which they are matched.

†Direct animal contact includes any of the following specific exposures: physical contact with animals or animal products (i.e. carcasses, bodily fluids, secretions, urine, excrement, or raw meat) in any setting (i.e., farm, livestock market, slaughterhouse, racetrack or stable, or other animal-related venues) or engaging in certain animal-related activities (i.e., feeding animals, cleaning their housing, slaughtering them, assisting with their birth, milking them, kissing or hugging them, or other related tasks).

‡Among persons who did not have direct contact with a specific animal, indirect animal contact includes any of the following exposures: having household (HH) members visit farms or markets where the animal is present, having the animal in or around the home, or visiting a location during the exposure period where the animal is kept, i.e., a farm, market, or slaughterhouse.

Technical Appendix Table 2. Selected demographic, exposure, and underlying condition information of MERS CoV cases with any direct contact with camels, Saudi Arabia, March 16–November 13, 2014.

	Demographic characteristics					During 14 days before illness onset								
ID	Nationality	Sex	Age, y	Owns farm	Camels at home	Visited farm	Camels on farm	Had contact with animals	Touched anything	Had contact with carcass	Contact with animal bedding	Fed animals	Cleaned animal housing	Cleaned farm equipment
1	Saudi	M	60	Yes	Yes	Yes	Yes	Yes	No	No	No	No	No	No
2	Saudi	M	64	Yes	Yes	Yes	Yes	Yes	Yes	No	No	No	No	No
3	Saudi	M	51	No	No	Yes	Yes	No	Yes	Yes	No	No	No	No
4*	Saudi	M	57	No	No	No								
5	Saudi	M	72	Yes	Yes	Yes	Yes	Yes	Yes	No	No	Yes	No	No
6	Saudi	M	53	Yes	Yes	Yes	Yes	Yes	No	No	No	Yes	No	No
7†	Saudi	M	49	Yes	Yes	Yes	Yes	Yes	Yes	Yes	Yes	Yes	Yes	Yes
8	Saudi	M	51	Yes	Yes	Yes	Yes	Yes	No	No	No	No	No	No
9	Saudi	M	68	No	No	Yes	Yes	Yes	No	No	Yes	Yes		No
10†	Saudi	M	48	Yes	Yes	Yes	Yes	Yes	Yes	No	Yes	Yes	Yes	Yes

*Blank cells indicate "not applicable" because this case-patient did not visit a farm.
†Blank indicate missing data.

	During 14 days before illness onset							Underlying condition/behavior			
ID	Slaughtered animals	Milked camels	Kissed/hugged camels	Visited livestock market	Visited slaughterhouse	Visited racetrack	Drank unpasteurized. camel milk	Diabetes	Emphysema	Heart disease	Current smoker
1	No	No	Yes	Yes	No	No	No	Yes	No	Yes	Yes
2	No	Yes	No	No	No	Yes	Yes	No	No	Yes	No
3	No	No	No	Yes	Yes	No	Yes	Yes	No	No	No
4				Yes	Yes	No	No	Yes	No	No	No
5	No	Yes	No	No	No	Yes	Yes	No	No	No	No
6	No	Yes	No	Yes	No	No	No	No	No	Yes	No
7		No	No	Yes		Yes	No		No	No	Yes
8	No	No	No	Yes	No	No	No	Yes	No	No	No
9	No	Yes	No	No	No	No	Yes	Yes	No	Yes	No
10	Yes	Yes		No	No	Yes	No	Yes	Yes	No	No
